# How Adolescents and Adults Learn About Changes in the Trustworthiness of Others Through Dynamic Interaction

**DOI:** 10.3389/fpsyg.2021.690494

**Published:** 2021-08-18

**Authors:** Siying Li, Xinmin Hao, Yueqi Mei, Yinyi Cheng, Nan Sun, Chen Qu

**Affiliations:** ^1^Key Laboratory of Brain, Cognition and Education Sciences, Ministry of Education, South China Normal University, Guangzhou, China; ^2^School of Psychology, South China Normal University, Guangzhou, China; ^3^Center for Studies of Psychological Application, South China Normal University, Guangzhou, China; ^4^Guangdong Key Laboratory of Mental Health and Cognitive Science, South China Normal University, Guangzhou, China; ^5^Guangdong Country Garden School, Foshan, China; ^6^School of Education, Guangzhou University, Guangzhou, China

**Keywords:** trust, trust game, adolescent, social learning, dynamic interaction, computational modeling

## Abstract

Whether to trust or distrust another individual is a complex interpersonal challenge, especially when such individuals behave inconsistently. It is still unclear as to how individuals learn and adapt to fluctuations in the trustworthiness of others and how this process changes from adolescence to adulthood. To address these issues, we implemented repeated rounds of a trust game within the context of a complicated and changeable interpersonal environment. Specifically, adolescents and adults played the role of trustors who had to decide whether to invest money in two anonymous partners carrying the risk of no reciprocation. Unbeknownst to participants, these two partners had different trustworthiness profiles: one partner initially yielded a higher initial return rate (70%) while the other initially yielded a lower initial return rate (30%). Crucially, over repeated rounds, these two partners gradually changed their responses to the point where, finally, return rates were both neutral (50%). Results indicated that all participants showed less updating in the negative direction in response to good-to-neutral partners while more updating in the positive direction in response to the bad-to-neutral partner. Compared to adults, this behavioral disparity in responses to good-to-neutral and bad-to-neutral partners was less pronounced in adolescents. Based on the computational modeling approach, the potential mechanisms underlying their behavioral patterns were revealed: the higher learning rate promoted flexible adaptions in participants to untrustworthy trustees as they changed to neutral. The less pronounced distinction between good-to-neutral and bad-to-neutral partners in adolescents was related to their lower learning rate. Overall, our study extends the understanding of trust behavior to a fluctuating social context and highlights the role of social learning in social emotion and interaction.

## Introduction

The importance of interpersonal trust within a well-functioning society is immeasurable. Theories and researches have done much to advance our understanding of interpersonal trust which is considered to be a complex social behavior (Bellucci et al., 2017; Krueger and Meyer-Lindenberg, 2019). It relies on the interplay of at least three different factors including risk preferences, social preferences, and beliefs about others’ trustworthiness (Coleman, 1990; Fehr, 2009; Bellucci et al., 2017). The first two factors have close links with the agent of trust behavior, i.e., the trustor, which have more influence on the baseline of trust behaviors. The main effect of trustee is reflected in the third factor which is emphasized in social learning theory. Social learning theory defined interpersonal trust as a generalized expectancy held by an individual or a group that the word, promise, verbal, or written statement of another individual or group can be relied upon (Rotter, 1967, 1971; Szcześniak et al., 2012). This “generalized expectancy” is related to beliefs about others’ trustworthiness implying the influence from trustee to trustor. In this study, inspired by social learning theory, we focused on the social learning processing that updates beliefs about trustees’ trustworthiness to optimize trust behaviors.

On the one hand, beliefs about others’ trustworthiness built through social priors. For example, a trustee’s identity, ethnicity, stereotyped image, facial appearance as well as the first impression, that they may can all affect an individual’s expectancy of trustworthiness about them (van’t Wout and Sanfey, 2008; Boero et al., 2009; Fouragnan et al., 2013; Yu et al., 2014; Cañadas et al., 2015; Lee et al., 2016; Bellucci et al., 2017; Telga et al., 2018). One study found that participants showed more trust-related behaviors towards partners with high social status who had made a promise compared with those of lower status who had made the same promise (Blue et al., 2020). Besides social status, the social relationship is also an important social prior that can influence trust behaviors as the following study. In an interactive trust game, participants acted as investors who were required to decide whether to share money with three trustees bearing distinct identities: a friend of the participant, an unacquainted confederate, and a nonsocial computer. Participants were shown to invest more frequently with their friends compared with the confederate and computer even they had equivalent reciprocation of collaborative decisions (Fareri et al., 2015). Human beings have a great ability to judge others’ trustworthiness rely on the social prior even with limits of time and information. It is found that people are able to evaluate trustworthiness based on the first impression of faces within a very short time (100 ms; Willis and Todorov, 2006). Besides the above characteristics of which influences formed through socialization in real experience, it can be learned in a laboratory environment. This was demonstrated by a study that manipulated fictional partners to appear to be either good, bad, or neutral at performing a Cyberball game just before a trust game. Results showed that even though there was no overlap in behavior between the Cyberball game and the trust game and their return rates were equal, participants invested less in the bad partner who rarely passed the ball to themselves in the Cyberball game compared with the other two partners (Fareri et al., 2012). Taken together, these findings corroborate the claim that prior-based knowledge plays a pivotal role in trust behavior.

On the other hand, in addition to prior-based knowledge, socializing with a partner in a trust-related activity is another way to estimate their trustworthiness. An fMRI study explored the influence of both prior-based and interaction-based learning on trustworthiness. In that context, if a cue representing trustworthy or untrustworthy was provided, participants would obtain prior-based knowledge of trustees’ trustworthiness before the trust game, while if no cues were provided, they had to learn trustees’ trustworthiness based on the interaction during the trust game (Fouragnan et al., 2013). This study demonstrated that reinforcement learning patterns reflected in behavior correlated with striatal activation only when participants had to estimate the level of partners’ trustworthiness without available prior. When prior knowledge was available, it oriented initial decisions and reflected in medial prefrontal cortex activity (Fouragnan et al., 2013). It is supported that the use of prior-based and interaction-based information in guiding trust behavior is implemented by different neural mechanisms.

In addition to the conflict between prior knowledge and current interaction, it is also possible to appear fluctuations for trustworthiness during the interaction. How people learn these fluctuations in trustworthiness from interaction has, until now, been unclear. In terms of changes in trustworthiness, a good or bad initial impression may act as two distinct learning reference frames which can then influence subsequent learning and updating processes, i.e., declining trustworthiness from a good initial frame, and increasing trustworthiness from a bad frame. In terms of impression formation, findings, based on diagnostic statements, showed that the influence of changes in the impression that shift from good to bad is greater than changes that shift from bad to good (Reeder and Coovert, 1986; Baumeister et al., 2001). With this disparity in mind, we can ask the following question. In natural interaction-based learning, how does the reference frame influence the process of learning? To investigate this, we employed a repeated trust game to create a situation that individuals learn to trust by evaluating feedbacks of repeated interactions.

Interaction-based trustworthiness learning embodies the social learning processing of interpersonal trust that is a main social emotion in daily interaction. These two key social functions mature gradually with development and socialization (Kilford et al., 2016) which led us to dig deep into this question from a developmental perspective. Development theory, supported by empirical evidence, has identified a close link between age and trust construction (Bernath and Feshbach, 1995; Kilford et al., 2016). Psychologists maintain that the construction of trust can be placed along a developing track throughout the human lifespan (Erikson, 1993, 1994; Sakai, 2010). One study examined how trust develops across time by recruiting participants ranging in age from eight years old to retirement age to play the trust game. They found that trust changed as a function of age from early childhood to early adulthood and then stayed constant during the rest of adulthood (Sutter and Kocher, 2007). The same pattern of age-related changes was also found with respect to reciprocity, the probability of returning money to trustors increases with age when participants as trustees in the trust game (van den Bos et al., 2010). An increase in trust behavior and trustworthiness was observed in lockstep with age. This phenomenon suggests that improvements in social function have close links with corresponding increases in socialization (van den Bos et al., 2010, 2011).

Although these findings offer important insights into age differences in trust behavior, they do not tell the whole story. When trustees show varied and changing levels of trustworthiness, we are yet to establish what role of age plays in the construction of trust. In this study, we explored this issue by examining both adolescents and adults to gain insights into their reaction patterns. From adolescence, individuals begin to experience a more complicated social life (Belli et al., 2012). Negative elements, which parents were previously able to filter out, become more commonplace than before. As adolescents, these individuals spend less time within an environment that is managed by parents and, as a consequence, they begin to face more mixed and changeable information. As such, adolescents need to begin to apply their own way of dealing with changeable situations and intricate relationships. At the same time, adolescents are experiencing important transitions across physical, social, behavioral, and cognitive domains and gradually they are moving to their adulthoods (Steinberg, 2005; Kilford et al., 2016). Thus, from adolescence to adulthood, this is a key period of social cognitive development. It is still unclear as to how these maturing adolescents learn about changes in the trustworthiness of others and deal with complicated interpersonal trust problems and what is the development trend in this issue from adolescence to adulthood. By studying both adolescents and adults, the second aim of the current study is to address this gap in the literature.

Taken together, this study highlights two key issues. The focus of this study was in part driven by the question of cognitive conflict in interaction-based learning: specifically whereby a trustworthy agent can become less trustworthy and an untrustworthy agent can become less untrustworthy over a period of interaction. In repeated rounds of the trust game, participants played as trustors against two anonymous trustees with changeable return rates. By preprogramming trustees’ return rates, we were able to track the behaviors of participants within two learning reference frames. That is a reference frame in which the partner reciprocated from 70 to 50% of the time, and another in which the partner reciprocated from 30 to 50% of the time. Next, we focused on the different behavioral patterns between adolescents and adults and compared their performance in the above dynamic interaction. The difference between these two age groups implies the development trend of reactive patterns to changeable interpersonal trust from adolescence to adulthood.

In addition, running repeated rounds of the trust game offered the prospect of gaining more insights through computational modeling. This approach has previously been used in studies to explore underlying mechanisms in the overt behaviors associated with a variety of social decision making and social learning phenomena (Fareri et al., 2012, 2015; van Baar et al., 2019). It is helpful to build an explanatory cognitive mechanism and a framework with which to predict behavioral performance (Konovalov et al., 2018; Lockwood and Klein-Flügge, 2020). In view of the advantages afforded by computational modeling, we used a reinforcement learning model with the aim of decoding trust construction processing in two reference frames across the two age groups.

## Materials and Methods

### Participants

The sample size was determined by two priori power analyses using G^*^power (Faul et al., 2007). One analysis was conducted for learning reference frame effect (one-sample t-test, two-tailed), it indicated a required sample size of *N* = 34 of each age group to be able to find an effect of at least *d* = 0.5 at *α* = 0.05 with a standard statistical power of 0.8. The other is for age effect (within-between interaction in repeated-measures ANOVA), at least 17 participants were required in each age group to be able to find an effect of at least *f* = 0.25 at *α* = 0.05 with a standard statistical power of 0.8. Considering possible exclusion, a total of 74 healthy participants were recruited from a university (adult sample) and a mainstream school (adolescent sample). Participants and their guardians (for the adolescent group) read the instruction and signed the informed consent. Two participants were excluded from analyses because one participant was interrupted during the experiment, and the other participant misunderstood the task. The remaining 72 participants were divided into two age groups: 36 adults (20 females, *M* = 21.86 years, SD *=* 2.09, range *=* 19–29) and 36 adolescents (19 females, *M* = 15.75 years, SD *=* 1.14, range *=* 14–18).

### Experimental Paradigm

**Figure 1:**
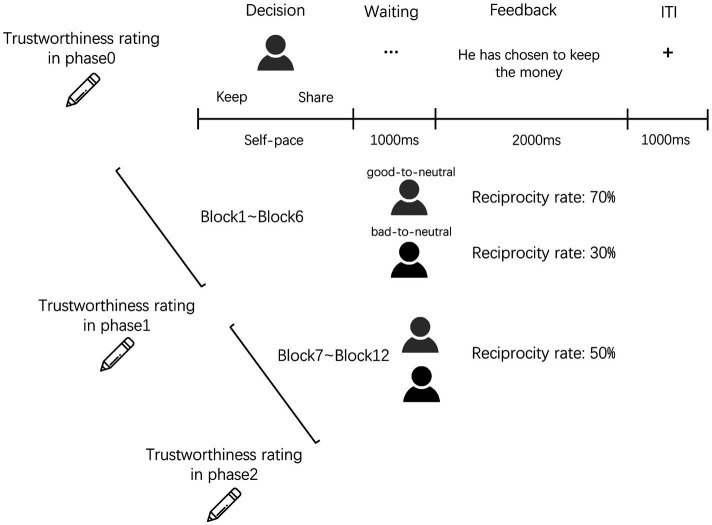
Experiment schematic. Participants completed a two-phase trust game interspersed with three trustworthiness ratings (i.e., phase0: before the trust game, phase1: at the end of Block6, phase2: at the end of Block12). Participants as trustors interacted with two trustees. The good-to-neutral trustee yielded a higher initial return rate (70%) while the bad-to-neutral trustee yielded a lower initial return rate (30%) in Block1 to 6. In Block7 to 12, these two trustees changed their return rates both to neutral (50%).

The experiment was conducted online by E-Prime 2.0. Following the preparation, participants completed a two-phase trust game interspersed with three trustworthiness ratings (Figure 1). We implemented the multishot binary version of the trust game in this study in which trustors were expected to do a binomial forced-choice, i.e., with an endowment by the experimenter, the trustor chose to keep all the money or share all the money to the trustee in each trial. The percentage of trials in which trustors shared on average across the experiment condition captures trust. This variation of the typical trust game and the operational definition of trust have been validated by many studies (Delgado et al., 2005; Evans and Krueger, 2010; Aimone and Houser, 2012, 2013; Fareri et al., 2012, 2015; Fouragnan et al., 2013; Cañadas et al., 2015). In this study, participants were informed that they would play as Player A (i.e., trustors) to interact with participants from earlier sessions of this experiment who played as Player B (i.e., trustees). They were required to decide whether to invest in two same-gender anonymous fictional partners (facial stimuli taken from the Chicago face database; Ma et al., 2015).

At the start of the trust game, participants firstly made a trustworthiness rating for each partner on a 9-point Likert scale (1*=*not at all, 9*=*a lot). In the following trust game, participants were endowed with 5 yuan before starting a trial. Each trial was composed of two stages, first step was to measure the trust in which the initiative was seized by the trustor. They needed to decide whether to share money with a trustee. A choice to share money was described as an investment, resulting in a tripling of the money to 15 yuan for the trustee on a given trial. If they decided to give money to a trustee, that trustee would obtain 15 yuan and triggered the second step. So that the trustee can decide whether to reciprocate the trustor. If the trustee decided to reciprocate, himself/herself and the trustor (participant) would gain half of the 15 yuan separately. Otherwise, the trustee may keep all of 15 yuan, meanwhile, the trustor would obtain nothing. If participants decided to keep the money in the first step, signaling the end of the trial in the first step, the trustee obtained nothing in that round. After participants made their decisions, they were presented with one of three possible feedbacks based on their responses: “You have kept the money,” “She/He has chosen to keep the money,” or “She/has chosen to share the money.” Before the experiment, participants were informed that one random trial would be selected at the end of the game, the outcome of this trial, i.e., obtaining 0, 5, or 7.5 yuan would as a reward for participants.

A total of 72 trials were included in the trust game, evenly distributed across twelve blocks. The first six blocks composed phase1 the other six blocks composed phase2. Thirty-six trials per partner condition were randomly administered across these blocks. The second trustworthiness rating was conducted after participants finished phase1. After participants finished the whole trust game, they rated the trustworthiness for the last time.

Unbeknownst to participants, trustees’ decisions in the second step did not control by other participants. Trustees had preprogrammed reciprocity rate in which participants chose to invest: good-to-neutral partner had a high initial reciprocity rate (phase1; 70%), bad-to-neutral had a low initial reciprocity rate (phase1; 30%), and as time goes by, in the later period, these two partners changed to the same neutral in reciprocity rate (phase2; 50%). The trial procedure and the task schematic were shown in Figure 1.

### Computational Modeling

#### Model Building

We employed computational models to test the possible link between internal cognitive mechanisms and trust behaviors. As an approach to the mechanism of learning, the reinforcement learning model applied to account for a wide range of learning behaviors including the social learning domain (King-Casas, 2005; Jones et al., 2011; Fareri et al., 2012, 2015; Kishida and Montague, 2012; Konovalov et al., 2018; Lockwood and Klein-Flügge, 2020). Based on the reinforcement learning frame and adapted for task context, we constructed three models, RW_P model, RW_LG model, and RW_PLG model and focused on an important free parameter, the learning rate.

These models are all formalized by decision theory which states that people make decisions to maximize their expected value. Expected value (*EV*) of investment to encountered partner *i* on trial *t* can be expressed as the likelihood of obtaining reward (7.5 yuan; in our context is the occurrence of partner’s reciprocation; Eq. 1).

The likelihood of receive reciprocation is learned information that needs to update in a trial-by-trial way. Computational models involving learning processing have their updating mechanism, that is, how to link between previous knowledge and novel knowledge or how new information integrates into prior knowledge to update the current stage. Reinforcement learning updates information through prediction error which refers to the difference between prediction and outcome. This difference facilitates the move from subjective belief to reality. We used a Rescorla–Wagner prediction error rule to update participants’ expectations (Sutton and Barto, 2012). Given the trial *t*, if participants invested to partner *i*, their new expectation of *Pi* will update based on the feedback (*γ* = 1, *Pi* reciprocate; *γ* = 0, *Pi* defect). The degree of updating or the weight on current prediction error was also influenced by learning rate α, this free parameter was bounded between 0 and 1. With the same prediction error, the higher α indicated the higher degree of updating would be integrated into the *P*(*t*+1) (see Eq. 3). This point varied in our three different models. In the RW_P model, we set different α for phase1 and phase2 separately for two partners on the basis of the hypothesis that given different volatility environments, different learning rates (*α_phase1_*, *α_phase2_*) can better represent the cognitive processing. Another consideration was the main different sense between loss (partners’ betrayal) and gain (partners’ reciprocation). In the RW_LG model, separate α was applied to loss and gain context (α_loss_, α_gain_) for these two partners, respectively, (Fareri et al., 2012, 2015). We also tested the possibility of combine influence with phase and loss/gain context in the RW_PLG model in which involved different learning rate both for phase and attribute of feedback (*α_loss_phase1_*, *α_loss_phase2_*, *α_gain_phase1_*, *α_gain_phase2_*).

The updated *P* (*t*+1) would generate an updated *EV* (*t*+1). It is transformed by the softmax function to calculate the probability of participants deciding to invest (*IPi*) the given partner *i* (see Eq. 2). The *β* in Eq. 2 was a free parameter that mirrored the extent of strategy changing. It bounded between 0 and 1, reflecting more explorative when it close to 1 whereas more exploitative when it close to 0. The probability of keep money equaled 1-*IPi*.

(1) $$EVi\left(t\right)=Pi\left(t\right)*\left(7.5\right)$$

(2) $$IPi=\frac{e^{\frac{EVi\left(t,1\right)}{\beta }}}{e^{\frac{EVi\left(t,1\right)}{\beta }}+e^{\frac{EVi\left(t,2\right)}{\beta }}}$$

(3) $$Pi\left(t+1\right)=Pi\left(t\right)+\alpha *\left(\gamma i\left(t\right)-Pi\left(t\right)\right)$$

(4) $$LLE=\sum_{t=1}^{n}\log\left(IPi,j\left(t\right)\right)$$

#### Model Estimation and Comparison

Log-likelihood estimation was calculated through maximizing function in Eq. 4 to estimate free parameters of each model for each participant, where *j* indexes the decision (share or keep), and *n* is the total number of trials.

For these three alternative models, we used the Akaike Information Criterion (AIC; Akaike, 1974), which applied a penalty scaled by the number of free parameters of a complicated model, to choose a more representative model. These estimations conducted using custom MATLAB scripts.

## Results

### Reference Frame Effect in Reciprocity Updating

**Figure 2:**
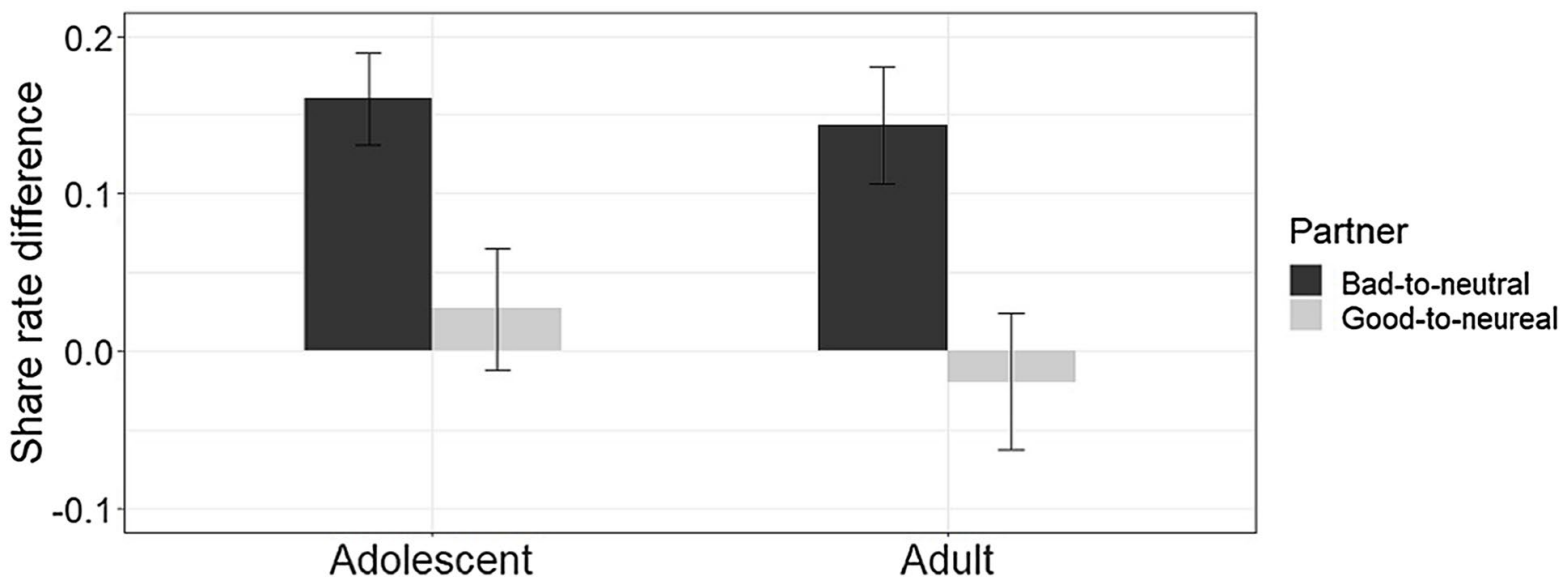
The updating of share rates. The percentage difference of trials in which adolescents and adults shared on average between phase1 and phase2 separately to good-to-neutral and bad-to-neutral partner (error bars reflect SE).

To examine the updating of reciprocity changes, we used one-sample t-tests on the share rate differences between phase1 and phase2 (i.e., the difference of percentage of the decision to share between phase1 and phase2) for two partners separately, by comparing the share rate differences with the neutral value zero in two age groups. The results showed that, in the adolescent group, to the good-to-neutral trustee, the value of the share rate of phase2 minus that of phase1 was not significantly different from zero (*M* = 0.03, SD *=* 0.23, *t*_(35)_ *=* 0.69, *p* = 0.49, *d* = 0.11). As to the bad-to-neutral trustee, adolescents’ share rate changed significantly (*M* = 0.16, SD *=* 0.17, *t*_(35)_ *=* 5.53, *p* < 0.001, *d* = 0.94). In the adult group, to the good-to-neutral trustee, the value of the share rate differences between the two phases was not significantly different from zero (*M* = −0.02, SD *=* 0.26, *t*_(35)_= −0.45, *p* = 0.65, *d* = 0.08). As to the bad-to-neutral trustee, adults changed their share rates significantly (*M* = 0.14, SD *=* 0.22, *t*_(35)_ *=* 3.84, *p* < 0.001, *d* = 0.64). These results reflected that for the good-to-neutral partner, all participants’ share rates did not change significantly in the two phases, while for the bad-to-neutral partner, all participants’ investments in phase2 were significantly higher than in phase1 (Figure 2).

### Age Difference in Trust Decisions

In order to explore the roles of age and partner’s return rate in the time course of the experiment, with Partner (good-to-neutral vs. bad-to-neutral) and Block (block1-block12) as within-subject variables and Age (adult vs. adolescent) as a between-subjects variable, a three-way repeated ANOVA was conducted on the percentages of decisions to share. Results indicated a significant main effect of Partner, *F*_(1, 70)_ *=* 39.68, *p* < 0.001, $\eta_{p}{}^{2}$ *=* 0.36. Participants’ share rates were significantly higher when they interacted with the good-to-neutral trustee (*M* = 0.62, SD *=* 0.02) than that of the bad-to-neutral trustee (*M* = 0.49, SD *=* 0.02). The main effect of Block was significant, *F*_(11, 770)_ *=* 4.41, *p* < 0.001, $\eta_{p}{}^{2}$ *=* 0.06, demonstrated that share rates changed with learning processing. Significant Partner × Block interaction was observed, *F*_(11,770)_ *=* 4.86, *p* < 0.001, $\eta_{p}{}^{2}$ *=* 0.07. As the trustees’ return rates changed, participants’ share rates also changed, and they learned different changes in return rates of two trustees during the experiment. We found a significant interaction of Age × Partner, *F*_(1, 70)_ *=* 5.68, *p* = 0.02, $\eta_{p}{}^{2}$ =0.08. Next, simple effects were tested. We examined the differences of investment rates between two age groups to the good-to-neutral partner and the bad-to-neutral partner separately. No significant difference was found between two age groups (good-to-neutral partner: *p* = 0.30; bad-to-neutral partner: *p* = 0.16). On the other side, we tested the differences of investment rates between two partners in adolescent group (good-to-neutral partner: *M* = 0.60, SD *=* 0.03; bad-to-neutral partner: *M* = 0.52, SD *=* 0.03; *F*_(1, 70)_ =7.67, *p* = 0.01, $\eta_{p}{}^{2}$ *=* 0.10) and adult group (good-to-neutral partner: M *=* 0.65, SD *=* 0.03; bad-to-neutral partner: *M* = 0.47, SD *=* 0.03; *F*_(1, 70)_ *=* 37.69, *p* < 0.001, $\eta_{p}{}^{2}$ *=* 0.35) separately. These two groups both showed significantly higher share rates to the good-to-neutral partner than the bad-to-neutral partner, indicated all participants learned the difference between two partners.

**Figure 3:**
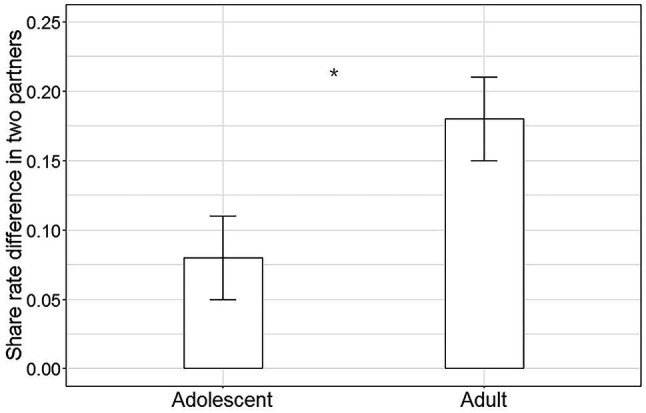
Age effect on the share rate difference. Adolescents’ percentage difference between trials in which shared with good-to-neutral and bad-to-neutral was significantly smaller than that of the adult group (error bars reflect SE, ^*^p < 0.05).

To search for the origin of the interaction between age and partner, we further compared two group participants’ share rate differences between two partners by independent-sample t-test (Figure 3). Results showed that share rate difference of adult was significantly higher than that of the adolescent (adult: *M* = 0.18, SD *=* 0.19; adolescent: *M* = 0.08, SD *=* 0.15; *t*_(70)_ *=* 2.46, *p* = 0.02, *d* = 0.58). Combined with the above findings, it is found that adults and adolescents both invested higher to the good-to-neutral partner than the bad-to-neutral partner, while adolescents’ share rate difference to these two partners was significantly smaller than that of adults which originated the significant interaction between age and partner.

As to reaction time, no significant main effect or interaction was found (*ps* > 0.05).

### Trustworthiness Rating Difference in the Trust Game

The trustworthiness ratings for each partner served as a different condition manipulation check. The trustworthiness ratings were entered into a 2 (Age: adult vs. adolescent) × 2 (Partner: good-to-neutral vs. bad-to-neutral) × 3 (Phase: phase0, phase1, phase2) repeated-measures ANOVA (“phase0” referred to the first trustworthiness rating of trustees at the start of the experiment) to investigate the trustworthy changing processing based on the influence of age, partner and phase in subjective feeling. We found the significant interaction of Age × Partner, *F*_(1,70)_ *=* 6.24, *p* = 0.02, $\eta_{p}{}^{2}$ *=* 0.08. *Post hoc* tests showed that adults (good-to-neutral: *M* = 5.05, SD *=* 0.19; bad-to-neutral: *M* = 4.02, SD *=* 0.19; *F*_(1,70)_ *=* 64.06, *p* < 0.001, $\eta_{p}{}^{2}$ *=* 0.48) and adolescents (good-to-neutral: *M* = 4.45, SD *=* 0.19; bad-to-neutral: *M* = 3.88, SD *=* 0.19; *F*_(1,70)_ *=* 19.99, *p* < 0.001, $\eta_{p}{}^{2}$ *=* 0.22) both rated good-to-neutral partner more trustworthy than bad-to-neutral partner. Adults placed more trust in the good-to-neutral partner than adolescents, *F*_(1,70)_ *=* 4.97, *p* = 0.03, $\eta_{p}{}^{2}$ *=* 0.07. The interaction of Partner× Phase was significant, *F*_(2, 140)_ *=* 50.17, *p* < 0.001, $\eta_{p}{}^{2}$ *=* 0.42. Further analysis suggested that, both in phase1 (*F*_(1,70)_ *=* 114.99, *p* < 0.001, $\eta_{p}{}^{2}$ *=* 0.62) and phase2 (*F*_(1,70)_ *=* 4.27, *p* = 0.04, $\eta_{p}{}^{2}$ *=* 0.06), the trustworthiness ratings for good-to-neutral partner (phase 0: *M* = 4.10, SD *=* 0.19; phase1: *M* = 5.81, SD *=* 0.16; phase2: *M* = 4.35, SD *=* 0.20) were significantly higher than that of bad-to-neutral partner (phase0: *M* = 4.25, SD *=* 0.19; phase1: *M* = 3.58, SD *=* 0.16; phase2: *M* = 4.01, SD *=* 0.19). And as to good-to-neutral partner, trustworthy scores of phase1 was significantly higher than that of phase0 and phase2, *F*_(2,140)_ *=* 41.42, *p* < 0.001, $\eta_{p}{}^{2}$ *=* 0.55. As to the bad-to-neutral partner, the trust score of phase1 was significantly lower than that of phase0 and phase2, *F*_(2,140)_ *=* 7.03, *p* < 0.001, $\eta_{p}{}^{2}$ *=* 0.17. We also found significant main effects of Partner (*F*_(1, 70)_ *=* 77.81, *p* < 0.001, $\eta_{p}{}^{2}$ =0.53) and of Phase (*F*_(2,140)_ *=* 5.80, *p* = 0.004, $\eta_{p}{}^{2}$ *=* 0.076). No other significant main effect or interaction was found (*ps >* 0.05).

### Revelation in the Computational Model

Results of model estimation and comparison were shown in Table 1. Nonparametric Wilcoxon signed-rank tests were conducted on the value of AIC of three models. The AIC value of the RW_LG model was significantly smaller than the other two models, indicating its better fitness on participants’ behaviors than the RW_P model (*z* = −7.06, *p* < 0.001, *dz* = 1.07) and RW_PLG model (*z* = −7.20, *p* < 0.001, *dz* = 1.88).

**Table 1:** Model parameters.

Model	Partner	*α _loss_* (SE)	*α _gain_* (SE)	*α _phase1_*(SE)	*α _phase2_*(SE)	*α _loss_phase1_*(SE)	*α _loss_phase2_*(SE)	*α _gain_phase1_*(SE)	*α _gain_phase2_*(SE)	*β* (SE)	AIC (SE)
RW_LG	Good-to-neutral	0.47(0.03)	0.15(0.03)	–	–	–	–	–	–	rowspan="2">0.96(0.02)	rowspan="2">62.74(2.36)
	Bad-to-neutral	0.57(0.03)	0.18(0.03)	–	–	–	–	–	–
RW_P	Good-to-neutral	–	–	0.12(0.03)	0.35(0.05)	–	–	–	–	rowspan="2">0.99(0.001)	rowspan="2">79.06(3.07)^***^
	Bad-to-neutral	–	–	0.56(0.04)	0.20(0.04)	–	–	–	–
RW_PLG	Good-to-neutral	–	–	–	–	0.52(0.04)	0.36(0.04)	0.19(0.04)	0.28(0.04)	rowspan="2">0.94(0.02)	rowspan="2">68.07(2.31)^***^
	Bad-to-neutral	–	–	–	–	0.61(0.03)	0.37(0.04)	0.27(0.04)	0.20(0.03)

*** *p* < 0.001; Comparison model: RW_LG.

**Figure 4:**
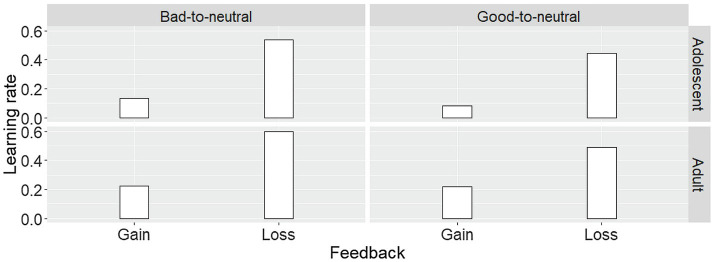
Learning rates in the reinforcement learning model (RW_LG model). Participants had a higher learning rate when they received feedbacks representing loss money than that of gain money. Compared with the good-to-neutral partner, the learning rate to the bad-to-neutral partner was higher. Adults’ learning rate was higher than adolescents’.

Further, in the framework of the RW_LG model, we tested whether different learning rates existed in different age groups. We conducted a repeated-measures ANOVA with Partner (good-to-neutral vs. bad-to-neutral) and Feedback (loss vs. gain) as within-subject variables and Age (adolescent vs. adult) as a between-subject variable on learning rates (Figure 4). It revealed that the main effect of Partner (*F*_(1,70)_ *=* 7.07, *p* = 0.01, $\eta_{p}{}^{2}$ *=* 0.09), the learning rate of bad-to-neutral partner was significantly higher than that of the good-to-neutral partner. The main effect of Feedback was significant (*F*_(1,70)_ *=* 113.10, *p* < 0.001, $\eta_{p}{}^{2}$ *=* 0.62), specifically the learning rate was higher when participants lost money than they gained. In addition, we also observed a significant main effect of Age (*F*_(1,70)_ *=* 4.31, *p* = 0.04, $\eta_{p}{}^{2}$ *=* 0.06), adult participants had a higher learning rate than adolescents.

## Discussion

In the present study, we ran repeated rounds of the trust game to explore how adolescents and adults learn about changes in trustworthiness through interaction. We recorded the trajectories of the trust decisions of participants as well as their trustworthy ratings. Moreover, we used computational modeling to delve into the mechanisms underlying the behaviors of participants. We designed our study such that two trustees who interacted with participants had preprogrammed patterns in their rates of return. One trustee exhibited a higher initial return rate while the other exhibited a lower initial return rate. After a period of time trustees changed to the point where both had reached a neutral rate of return. When interacted with two changeable trustees, we found that adolescents and adults showed similarities as well as differences in their patterns of behavior: (1) In both groups, we observed asymmetric patterns of trust behavior as a function of the two learning reference frames. Participants showed similar rates of trust towards the good-to-neutral partner between phase1 and phase2, thus showing leniency to the partner who reduced their reciprocity from phase1 to phase2. Participants increased their trust behaviors upon observing an increase in reciprocity in the bad-to-good partner. (2) Compared with adults, adolescents showed a minor distinction in their rates of trust between good-to-neutral and bad-to-neutral partners. (3) In the reinforcement learning model, findings of differences in learning rates to two partners and between two age groups provided potential reasons separately for observed asymmetric patterns in trust behaviors and the age differences.

### Asymmetrical Learning Patterns Produced by Two Changing Trends in Trustworthiness

One of the questions that we were most interested in was how participants respond to two partners with two distinct learning reference frames: one partner with a trustworthy to neutral frame and the other with an untrustworthy to neutral frame. To this aim, we implemented a two-phase task across repeated rounds of the trust game. In phase1, participants formed a trustworthy impression of one partner by observing a high return rate and formed an untrustworthy impression of another partner by observing a low return rate. In phase2, behavioral patterns changed such that participants observed a medium return rate from both partners. We found that, for good-to-neutral partners, a change in reciprocity from high to neutral did not result in a decline in investment. In contrast, we found that, for bad-to-neutral partners, a change in reciprocity from low to neutral led to increases in investment. Such asymmetrical learning patterns brought about by these two changing trends in trustworthiness are indicative of the effect of reference frames. In this instance, we observed less updating when reciprocity trended in a negative direction (good-to-neutral) in a good frame while more updating occurred when reciprocity trended in a positive direction (bad-to-neutral) in a bad frame.

However, at first glance, this result seemed difficult to reconcile with the main findings in the domain of impression formation, much of which stressed that bad impressions are more powerful and more resistant to disconfirmation than good ones (Skowronski and Carlston, 1989; Baumeister et al., 2001; Reeder and Coovert, 1986). Employing narrative statements of extreme and rare behaviors (theft or violence) that many studies used can formulate unshakeable bad impressions (Baumeister et al., 2001; Ferguson et al., 2019). At least to a certain extent, the diagnostic characteristics of negative information are due to their relative perceived frequency, these less frequently observed behaviors are weighted more heavily during evaluation (Mende-Siedlecki et al., 2013). However, in the present study, participants learned only through rounds of interactions, thus allowing more space for participants to evaluate and adapt to changes in partners. Such an asymmetrical learning pattern found in this research, whereby bad-to-neutral reciprocity induced more updating in impression learning solely through interactions, suggests that unlike social prior obtained by diagnostic statements interaction-based information influences the learning process through a different mechanism. In line with our findings, another study, in which participants learned about the characters of others by observing their choices in a trial-by-trial way, found that beliefs about bad people are volatile compared with beliefs about good people which are more stable (Siegel et al., 2018). By modeled participants’ choices with a Bayesian learning model, they observed a cognitive updating mechanism that was more flexible to bad information when the initial bad impression turns out to be inaccurate (Siegel et al., 2018). These findings of the framing effect imply different mechanisms that may be employed by interaction-based and prior-based cognitive updating processing. Based on previous studies, prior-based knowledge may be the main resource of “top-down processing” reflected in medial prefrontal cortex activity (Fouragnan et al., 2013; Krueger and Meyer-Lindenberg, 2019). In the communication between prior-based and interaction-based mechanisms, prior-based knowledge can diminish reliance on instant interaction-based information in the neural circuitry of trial-and-error reward learning (Delgado et al., 2005; Fouragnan et al., 2013; Krueger and Meyer-Lindenberg, 2019). The different behavioral expressions of framing effect in information updating based on social prior and interaction, for example, the current result and previous findings on diagnostic information, may originate from these two different cognitive mechanisms.

It is noteworthy that the results of subjective trust ratings did not show such an asymmetric pattern in these two frames. Specifically, participants correctly reported the changes in return rate produced by both partners, but chose not to change their trust rates to the good-to-neutral partner, and chose to forgive the bad-to-neutral partner. This particular pattern of results may be related to the type of task that we employed. In a cooperative background, like the trust game, choosing to cooperate is profitable. In another cooperative paradigm, the repeated prisoner’s dilemmas, participants seemed to forgive their partners who betrayed them once and decided to cooperate with them again (Fudenberg et al., 2012). In these cases, or similar reality, although detecting negative changes and signs of the bad character of others has significant meaning for living while prematurely set boundaries between good and bad just relying on limited information may miss out much potential benefit of cooperation in the future (Molander, 1985; Johnson et al., 2013; Siegel et al., 2018). Evolutionary models also found that these “forgive” strategy much better than end cooperation after a single betrayal (Wu and Axelrod, 1995; Fudenberg et al., 2012). This kind of strategic adaption is in line with our observations of how participants responded to good-to-neutral partners, in that they continued to cooperate with them even when they knew that they had become less trustworthy.

Taken together, we observed an asymmetrical pattern of behavioral updating whereby participants adapted their decisions regarding trustees who were either good-to-neutral or bad-to-neutral as a consequence of non-diagnostic interaction. Combined with previous studies, it implies that both the attribute of information (diagnostic and non-diagnostic) and the source of information (prior-based or interaction-based) play pivotal and distinct roles in the formation of impressions and the subsequent updating of behavior.

### Age Difference in the Trust Game

Our study highlights the prominent role played by developmental factors in trust behavior. Compared with the adult group, adolescent participants showed smaller differences in their patterns of trust behavior for good-to-neutral and bad-to-neutral partners. In other words, although they treated the two partners in distinct ways, the degree of distinction, as indexed by the amount invested, was not as pronounced as that produced by the adult group.

The more ambiguous tendency of the adolescents towards the two partners suggests a wait-and-see attitude. During adolescence, the demands of individuals for interaction with others increase progressively (Blakemore, 2008; Fouragnan et al., 2013). Given this propensity for increased interaction, one explanation for such a wait-and-see attitude could be a strategy to ensure more social attachments in the future. Another possible explanation is that the more limited social experiences of adolescents led to less rapid discrimination of the changing trends between the two agents. In this way, their less developed social functions may hamper the processing of social learning (Blakemore, 2008; Steinberg, 2008; Crone and Dahl, 2012; Kilford et al., 2016). We tested this possible explanation using the following modeling analysis.

### Explaining Underlying Trust Processes Using Reinforcement Learning Models

Next, we sought clues regarding the cognitive mechanisms underlying our observations of an asymmetrical updating pattern in trust behavior and the related age difference outlined above. We employed a computational modeling approach to further explore behavior within the context of a repeated trust game (Delgado et al., 2005; Fareri et al., 2012, 2015).

We formalized models using a reinforcement learning framework. Given the outcome that the RW_LG model fit the behaviors of participants significantly better than the other two candidates, we used it to explain our findings. In this model, based on the premise of benefit maximization, participants updated their predictions which then guided their behaviors and decisions. This assumption of decision rule originated in behavioral economics and assumes that humans select actions to maximize their projected utility (i.e., the expected value) (Fehr and Krajbich, 2014; Konovalov et al., 2018). This approach contributes to characterize how different motives and context factors influence behaviors by specifying the processing that individuals transfer relevant experimental variables into the expected value (Konovalov et al., 2018). Although the monetary reward is a goal that mankind pursues, their desire more than that. Humans are naturally social creatures, some social factors such as morality, equity, affect are valuable for them. Thus, decision theory was developed to suit human nature by taking abstract social value into expected value (Handgraaf et al., 2003; Hsu et al., 2008; Zhong et al., 2016). Though this decision rule is insufficient to explain all social behavior, its principle fits the current experiment well which can capture the effect of the changeable trustworthiness of trustees (Bellucci et al., 2017; Krueger and Meyer-Lindenberg, 2019). Another important mechanism in the computational model is the updating rule which is based on the prediction error in this study (O’Doherty et al., 2017). Prediction errors, which reflect the difference between a prediction about an outcome and reality (from feedbacks), play an integral role in learning processing and have been well established through numerous studies across domains and methods (Schultz, 2007, 2013; Behrens et al., 2009; Lockwood and Klein-Flügge, 2020). How much the degree of prediction error will be taken into internal computation is also restricted and scaled by another free parameter, named learning rate (Lockwood and Klein-Flügge, 2020). Learning rates vary among participants, reflecting the extent of learning through prediction error. In this study, we placed an emphasis on the learning rate and explored the function of learning rates in relation to decisions about trust.

We found significant differences in learning rates as a function of the two partners. Participants showed a higher learning rate when they interacted with bad-to-neutral trustees compared to that of good-to-neutral trustees. In addition, we observed significant differences in learning rates between adolescents and adults. Compared with adolescents, adults showed higher learning rates. These higher or lower learning rates do not necessarily have absolute links with worse or better performance, but rather depend on context. Learners with high learning rates are more likely to strongly update based on recent feedback (Lockwood and Klein-Flügge, 2020). This tendency may allow these learners to flexibly adapt to a changeable environment. Learners with low learning rates on the other hand are increasingly influenced by long-lasting previous feedbacks (Lockwood and Klein-Flügge, 2020).

The observed differences in the learning rate reconcile with our findings above. Firstly, the higher learning rate associated with bad-to-neutral partners prompted increasingly adaptive decisions compared with those for good-to-neutral partners. In other words, participants placed greater weight on initial information when updating behaviors in response to good-to-neutral trustees. Conversely, when these individuals interacted with bad-to-neutral partners, they adapted their decisions based more on current feedbacks. This implied close links between trustworthiness learning and asymmetrical patterns of two trends of trustworthiness changing. Secondly, with higher learning rates, the adult group was found to flexibly adapt their decisions. Adults showing a more pronounced distinction in behavioral patterns in response to two changeable partners compared to the adolescent group. Differences in learning rates between adolescents and adults may be an underlying explanatory factor for our observed differences in trust behavior. A weakened distinction between good-to-neutral and bad-to-neutral partners in adolescents may be associated with their reduced cognitive updating. Our results of computational modeling provided evidence for an ambiguous tendency in adolescents. Their weak updating, which may be a manifestation of their incomplete social experiences, may hamper this group from rapidly discriminating between two agents.

## Conclusion

These findings offer insights into how individuals update their representations of trust during instances of non-diagnostic interaction. They also serve to demonstrate the effect of age differences on trust behavior grounded in a more complicated and fluctuating social context. In addition, we provide a computational explanation as to how two changing trends in trustworthiness can produce the asymmetrical patterns in learning that were observed in this study. We were also able to reveal more insights as to why adult and adolescent groups showed different patterns of behavioral responses in the context of trust construction. We found that higher learning rates in relation to bad-to-neutral partners promoted rapid behavioral updating. When rates of learning were lower, participants kept investments high for good-to-neutral partners despite drops in trustworthiness. The relatively lower learning rates demonstrated in adolescents were associated with their weakened ability to distinguish between good-to-neutral partners and bad-to-neutral partners. Our study extends understanding of trust behavior to a fluctuating social context and explains behavioral differences brought by learning reference frames and developmental factors in a social learning perspective.

## Data Availability Statement

The raw data supporting the conclusions of this article will be made available by the authors, without undue reservation.

## Ethics Statement

The studies involving human participants were reviewed and approved by Human Research Ethics Committee of South China Normal University. Written informed consent to participate in this study was provided by the participants’ legal guardian/next of kin.

## Author Contributions

SL and CQ conceived and designed this study. SL programmed the experiments. XH, YM, and YC performed the experiments. SL and XH analyzed, interpreted the data, and wrote the manuscript. NS and CQ revised the manuscript. All authors contributed to the article and approved the submitted version.

## Conflict of Interest

The authors declare that the research was conducted in the absence of any commercial or financial relationships that could be construed as a potential conflict of interest.

## Publisher’s Note

All claims expressed in this article are solely those of the authors and do not necessarily represent those of their affiliated organizations, or those of the publisher, the editors and the reviewers. Any product that may be evaluated in this article, or claim that may be made by its manufacturer, is not guaranteed or endorsed by the publisher.

## References

[ref1] Aimone J. A. Houser D. (2012). What you don't know won't hurt you: a laboratory analysis of betrayal aversion. Exp. Econ. 15, 571–588. 10.1007/s10683-012-9314-z

[ref2] Aimone J. A. Houser D. (2013). Harnessing the benefits of betrayal aversion. J. Econ. Behav. Organ. 89, 1–8. 10.1016/j.jebo.2013.02.001

[ref3] Akaike H. (1974). A new look at statistical model identification. IEEE Trans. Autom. Control. 19, 716–723. 10.1109/TAC.1974.1100705

[ref4] Baumeister R. F. Bratslavsky E. Finkenauer C. Vohs K. D. (2001). Bad is stronger than good. Rev. Gen. Psychol. 5, 323–370. 10.1037/1089-2680.5.4.323

[ref5] Behrens T. E. J. Hunt L. T. Rushworth M. F. S. (2009). The computation of social behavior. Science 324, 1160–1164. 10.1126/science.1169694, PMID: 19478175

[ref6] Belli S. R. Rogers R. D. Lau J. Y. F. (2012). Adult and adolescent social reciprocity: experimental data from the trust game. J. Adolesc. 35, 1341–1349. 10.1016/j.adolescence.2012.05.004, PMID: 22691532

[ref7] Bellucci G. Chernyak S. V. Goodyear K. Eickhoff S. B. Krueger F. (2017). Neural signatures of trust in reciprocity: a coordinate-based meta-analysis. Hum. Brain Mapp. 38, 1233–1248. 10.1002/hbm.23451, PMID: 27859899 PMC5441232

[ref8] Bernath M. S. Feshbach N. D. (1995). Children’s trust: theory, assessment, development, and research directions. Appl. Prev. Psychol. 4, 1–19. 10.1016/S0962-1849(05)80048-4

[ref9] Blakemore S.-J. (2008). The social brain in adolescence. Nat. Rev. Neurosci. 9, 267–277. 10.1038/nrn2353, PMID: 18354399

[ref10] Blue P. R. Hu J. Peng L. Yu H. Liu H. Zhou X. (2020). Whose promises are worth more? How social status affects trust in promises. Eur. J. Soc. Psychol. 50, 189–206. 10.1002/ejsp.2596

[ref11] Boero R. Bravo G. Castellani M. Squazzoni F. (2009). Reputational cues in repeated trust games. J. Socio-Econ. 38, 871–877. 10.1016/j.socec.2009.05.004

[ref12] Cañadas E. Rodríguez-Bailón R. Lupiáñez J. (2015). The effect of social categorization on trust decisions in a trust game paradigm. Front. Psychol. 6:1568. 10.3389/fpsyg.2015.01568, PMID: 26528221 PMC4600900

[ref13] Coleman J. (1990). Foundations of Social Theory. Cambridge, MA: The Belknap Press of Harvard University.

[ref14] Crone E. A. Dahl R. E. (2012). Understanding adolescence as a period of social–affective engagement and goal flexibility. Nat. Rev. Neurosci. 13, 636–650. 10.1038/nrn3313, PMID: 22903221

[ref15] Delgado M. R. Frank R. H. Phelps E. A. (2005). Perceptions of moral character modulate the neural systems of reward during the trust game. Nat. Neurosci. 8, 1611–1618. 10.1038/nn1575, PMID: 16222226

[ref16] Erikson E. H. (1993). Childhood and Society. New York: W. W. Norton & Company Press.

[ref17] Erikson E. H. (1994). Identity: Youth, and Crisis. New York: W. W. Norton & Company Press.

[ref18] Evans A. M. Krueger J. I. (2010). The psychology (and economics) of trust. Social. Personality. Psychol. Compass. 3, 1003–1017. 10.1111/j.1751-9004.2009.00232.x

[ref19] Fareri D. S. Chang L. J. Delgado M. R. (2012). Effects of direct social experience on trust decisions and neural reward circuitry. Front. Neurosci. 6:148. 10.3389/fnins.2012.00148, PMID: 23087604 PMC3472892

[ref20] Fareri D. S. Chang L. J. Delgado M. R. (2015). Computational substrates of social value in interpersonal collaboration. J. Neurosci. 35, 8170–8180. 10.1523/JNEUROSCI.4775-14.2015, PMID: 26019333 PMC4444540

[ref21] Faul F. Erdfelder E. Lang A. G. Buchner A. (2007). G^*^ power 3: a flexible statistical power analysis program for the social, behavioral, and biomedical sciences. Behav. Res. Methods 39, 175–191. 10.3758/BF03193146, PMID: 17695343

[ref22] Fehr E. (2009). On the economics and biology of trust. J. Eur. Econ. Assoc. 7, 235–266. 10.1162/JEEA.2009.7.2-3.235

[ref23] Fehr E. Krajbich I. (2014). “Social preferences and the brain,” Neuroeconomics. 2nd *Edn*. eds. Glimcher P. W. Fehr E. (Elsevier: Academic Press), 193–218.

[ref24] Ferguson M. J. Mann T. C. Cone J. Shen X. (2019). When and how implicit first impressions can be updated. Curr. Dir. Psychol. Sci. 28, 331–336. 10.1177/0963721419835206

[ref25] Fouragnan E. Chierchia G. Greiner S. Neveu R. Avesani P. Coricelli G. (2013). Reputational priors magnify striatal responses to violations of trust. J. Neurosci. 33, 3602–3611. 10.1523/JNEUROSCI.3086-12.2013, PMID: 23426687 PMC6619519

[ref26] Fudenberg D. Rand D. G. Dreber A. (2012). Slow to anger and fast to forgive: cooperation in an uncertain world. Am. Econ. Rev. 102, 720–749. 10.1257/aer.102.2.720

[ref27] Handgraaf M. Dijk E. V. Cremer D. D. (2003). Social utility in ultimatum bargaining. Soc. Justice Res 16, 263–283. 10.1023/A:1025940829543

[ref28] Hsu M. Anen C. Quartz S. R. (2008). The right and the good: distributive justice and neural encoding of equity and efficiency. Science 320, 1092–1095. 10.1126/science.1153651, PMID: 18467558

[ref29] Johnson D. D. P. Blumstein D. T. Fowler J. H. Haselton M. G. (2013). The evolution of error: error management, cognitive constraints, and adaptive decision-making biases. Trends Ecol. Evol. 28, 474–481. 10.1016/j.tree.2013.05.014 23787087

[ref30] Jones R. M. Somerville L. H. Li J. Ruberry E. J. Libby V. Glover G.. (2011). Behavioral and neural properties of social reinforcement learning. J. Neurosci. 31, 13039–13045. 10.1523/JNEUROSCI.2972-11.2011, PMID: 21917787 PMC3303166

[ref31] Kilford E. J. Garrett E. Blakemore S.-J. (2016). The development of social cognition in adolescence: an integrated perspective. Neurosci. Biobehav. Rev. 70, 106–120. 10.1016/j.neubiorev.2016.08.016 27545755

[ref32] King-Casas B. (2005). Getting to know you: reputation and trust in a two-person economic exchange. Science 308, 78–83. 10.1126/science.1108062, PMID: 15802598

[ref33] Kishida K. T. Montague P. R. (2012). Imaging models of valuation during social interaction in humans. Biol. Psychiatry. 72, 93–100. 10.1016/j.biopsych.2012.02.037, PMID: 22507699 PMC3544196

[ref34] Konovalov A. Hu J. Ruff C. C. (2018). Neurocomputational approaches to social behavior. Curr. Opin. Psychol. 24, 41–47. 10.1016/j.copsyc.2018.04.009, PMID: 29738891

[ref35] Krueger F. Meyer-Lindenberg A. (2019). Toward a model of interpersonal trust drawn from neuroscience, psychology, and economics. Trends in Neuroences 42, 92–101. 10.1016/j.tins.2018.10.004, PMID: 30482606

[ref36] Lee N. C. Jolles J. Krabbendam L. (2016). Social information influences trust behaviour in adolescents. J. Adolesc. 46, 66–75. 10.1016/j.adolescence.2015.10.021, PMID: 26599529

[ref37] Lockwood P. Klein-Flügge M. (2020). Computational modelling of social cognition and behaviour-a reinforcement learning primer. Soc. Cogn. Affect. Neurosci. nsaa040. 10.1093/scan/nsaa040, PMID: 32232358 PMC8343561

[ref38] Ma D. S. Correll J. Wittenbrink B. (2015). The Chicago face database: A free stimulus set of faces and norming data. Behav Res. 47, 1122–1135. 10.3758/s13428-014-0532-5 25582810

[ref39] Mende-Siedlecki P. Baron S. G. Todorov A. (2013). Diagnostic value underlies asymmetric updating of impressions in the morality and ability domains. J. Neurosci. 33, 19406–19415. 10.1523/JNEUROSCI.2334-13.2013, PMID: 24336707 PMC6618766

[ref40] Molander P. (1985). The optimal level of generosity in a selfish, uncertain environment. J. Confl. Resolut. 29, 611–618. 10.1177/0022002785029004004

[ref41] O’Doherty J. P. Cockburn J. Pauli W. M. (2017). Learning, reward, and decision making. Annu. Rev. Psychol. 68, 73–100. 10.1146/annurev-psych-010416-044216, PMID: 27687119 PMC6192677

[ref42] Reeder G. D. Coovert M. D. (1986). Revising an impression of morality. Soc. Cogn. 4, 1–17. 10.1521/soco.1986.4.1.1

[ref43] Rotter J. B. (1967). A new scale for the measurement of interpersonal trust. J. Pers. 35, 651–665. 10.1111/j.1467-6494.1967.tb01454.x, PMID: 4865583

[ref44] Rotter J. B. (1971). Generalized expectancies for interpersonal trust. Am. Psychol. 26, 443–452. 10.1037/h0031464

[ref45] Sakai A. (2010). “Children’s sense of trust in significant others: genetic versus environmental contributions and buffer to life stressors,” in Interpersonal Trust During Childhood and Adolescence. ed. Rotenberg K. J. (Cambridge: Cambridge University Press), 56–84.

[ref46] Schultz W. (2007). Behavioral dopamine signals. Trends Neurosci. 30, 203–210. 10.1016/j.tins.2007.03.007, PMID: 17400301

[ref47] Schultz W. (2013). Updating dopamine reward signals. Curr. Opin. Neurobiol. 23, 229–238. 10.1016/j.conb.2012.11.012, PMID: 23267662 PMC3866681

[ref48] Siegel J. Z. Mathys C. Rutledge R. B. Crockett M. J. (2018). Beliefs about bad people are volatile. Nat. Hum. Behav. 2, 750–756. 10.1038/s41562-018-0425-1, PMID: 31406285

[ref001] Skowronski J. J. Carlston D. E. (1989). Negativity and extremity biases in impression formation: A review of explanations. Psychol. Bull. 105, 131–142. 10.1037/0033-2909.105.1.131

[ref49] Steinberg L. (2005). Cognitive and affective development in adolescence. Trends Cogn. Sci. 9, 69–74. 10.1016/j.tics.2004.12.005, PMID: 15668099

[ref50] Steinberg L. (2008). A social neuroscience perspective on adolescent risk-taking. Dev. Rev. 28, 78–106. 10.1016/j.dr.2007.08.002, PMID: 18509515 PMC2396566

[ref51] Sutter M. Kocher M. G. (2007). Trust and trustworthiness across different age groups. Games. Econ. Behav. 59, 364–382. 10.1016/j.geb.2006.07.006

[ref52] Sutton R. S. Barto A. G. (2012). Reinforcement Learning: An Introduction. London: The MIT Press.

[ref53] Szcześniak M. Colaço M. Rondón G. (2012). Development of interpersonal trust among children and adolescents. Pol. Psychol. Bull. 43, 50–58. 10.2478/v10059-012-0006-5

[ref54] Telga M. de Lemus S. Cañadas E. Rodríguez-Bailón R. Lupiáñez J. (2018). Category-based learning about deviant outgroup members hinders performance in trust decision making. Front. Psychol. 9:1008. 10.3389/fpsyg.2018.01008, PMID: 29977214 PMC6021525

[ref55] van Baar J. M. Chang L. J. Sanfey A. G. (2019). The computational and neural substrates of moral strategies in social decision-making. Nat. Commun. 10:1483. 10.1038/s41467-019-09161-6, PMID: 30940815 PMC6445121

[ref56] van den Bos W. van Dijk E. Westenberg M. Rombouts S. A. R. B. Crone E. A. (2011). Changing brains, changing perspectives: the neurocognitive development of reciprocity. Psychol. Sci. 22, 60–70. 10.1177/0956797610391102, PMID: 21164174

[ref57] van den Bos W. Westenberg M. van Dijk E. Crone E. A. (2010). Development of trust and reciprocity in adolescence. Cogn. Dev. 25, 90–102. 10.1016/j.cogdev.2009.07.004

[ref58] van’t Wout M. Sanfey A. G. (2008). Friend or foe: the effect of implicit trustworthiness judgments in social decision-making. Cognition 108, 796–803. 10.1016/j.cognition.2008.07.002 18721917

[ref59] Willis J. Todorov A. (2006). First impressions: making up your mind after a 100-ms exposure to a face. Psychol. Sci. 17, 592–598. 10.1111/j.1467-9280.2006.01750.x, PMID: 16866745

[ref60] Wu J. Axelrod R. (1995). How to cope with noise in the iterated prisoner’s dilemma. J. Confl. Resolut. 39, 183–189. 10.1177/0022002795039001008

[ref61] Yu M. Saleem M. Gonzalez C. (2014). Developing trust: first impressions and experience. J. Econ. Psychol. 43, 16–29. 10.1016/j.joep.2014.04.004

[ref62] Zhong S. Chark R. Hsu M. Chew S. H. (2016). Computational substrates of social norm enforcement by unaffected third parties. NeuroImage 129, 95–104. 10.1016/j.neuroimage.2016.01.040, PMID: 26825438 PMC5080368

